# CDFAN: Cross-Domain Fusion Attention Network for Pansharpening

**DOI:** 10.3390/e27060567

**Published:** 2025-05-27

**Authors:** Jinting Ding, Honghui Xu, Shengjun Zhou

**Affiliations:** 1 School of Information and Electrical Engineering, Hangzhou City University, Hangzhou 310015, China; dingjt@hzcu.edu.cn; 2 School of Computer Science and Technology, Zhejiang University of Technology, Hangzhou 310023, China; xhh@zjut.edu.cn; 3 Zhejiang Academy of Agricultural Sciences, Hangzhou 310021, China

**Keywords:** remote sensing, pansharpening, discrete wavelet transform, attention, information theory, mutual information

## Abstract

Pansharpening provides a computational solution to the resolution limitations of imaging hardware by enhancing the spatial quality of low-resolution hyperspectral (LRMS) images using high-resolution panchromatic (PAN) guidance. From an information-theoretic perspective, the task involves maximizing the mutual information between PAN and LRMS inputs while minimizing spectral distortion and redundancy in the fused output. However, traditional spatial-domain methods often fail to preserve high-frequency texture details, leading to entropy degradation in the resulting images. On the other hand, frequency-based approaches struggle to effectively integrate spatial and spectral cues, often neglecting the underlying information content distributions across domains. To address these shortcomings, we introduce a novel architecture, termed the Cross-Domain Fusion Attention Network (CDFAN), specifically designed for the pansharpening task. CDFAN is composed of two core modules: the Multi-Domain Interactive Attention (MDIA) module and the Spatial Multi-Scale Enhancement (SMCE) module. The MDIA module utilizes discrete wavelet transform (DWT) to decompose the PAN image into frequency sub-bands, which are then employed to construct attention mechanisms across both wavelet and spatial domains. Specifically, wavelet-domain features are used to formulate query vectors, while key features are derived from the spatial domain, allowing attention weights to be computed over multi-domain representations. This design facilitates more effective fusion of spectral and spatial cues, contributing to superior reconstruction of high-resolution multispectral (HRMS) images. Complementing this, the SMCE module integrates multi-scale convolutional pathways to reinforce spatial detail extraction at varying receptive fields. Additionally, an Expert Feature Compensator is introduced to adaptively balance contributions from different scales, thereby optimizing the trade-off between local detail preservation and global contextual understanding. Comprehensive experiments conducted on standard benchmark datasets demonstrate that CDFAN achieves notable improvements over existing state-of-the-art pansharpening methods, delivering enhanced spectral–spatial fidelity and producing images with higher perceptual quality.

## 1. Introduction

High-resolution multispectral (HRMS) imaging is essential for various domains [1,2,3,4,5,6], including environmental surveillance [7,8] and urban development [9,10]. However, due to constraints in satellite sensor technology, acquired data often consist of low-resolution multispectral (LRMS) images and high-resolution panchromatic (PAN) images [11,12,13,14]. To overcome this limitation, pansharpening techniques are employed to synthesize HRMS images by integrating the spectral richness of LRMS images with the fine spatial details of PAN images, thereby improving both spectral and spatial fidelity. From an information-theoretic perspective, the goal of pansharpening is to maximize the mutual information between PAN and LRMS sources while minimizing information loss and redundancy in the fused result. The quality of fusion can be interpreted through entropy-based metrics that evaluate information preservation and distortion.

In the early development of pansharpening, traditional methodologies dominated, including component substitution (CS) [15,16], multiresolution analysis (MRA)-based approaches [17,18], and model-driven techniques [19,20,21]. CS methods fused spatial details from high-resolution PAN images into corresponding LRMS images, while MRA-based strategies employed multi-scale fusion to capture hierarchical features. In contrast, model-based approaches formulated pansharpening as an inverse problem: the LRMS image was treated as a spatially degraded version of the HRMS image, whereas the PAN image was its spectrally degraded counterpart. The HRMS image was then reconstructed by solving this degradation model. However, the ill-posed nature of this formulation introduced significant challenges. To mitigate these issues, constraints based on sparsity, low-rank structures [22,23], and total variation [24] were incorporated to regularize the solution space, improving fidelity. While these methods proved effective in certain scenarios, their reliance on handcrafted features limited adaptability. Moreover, their inability to automatically capture complex spectral–spatial dependencies constrained their performance, particularly in handling fine-grained structures and high-frequency details.

**Figure 1:**
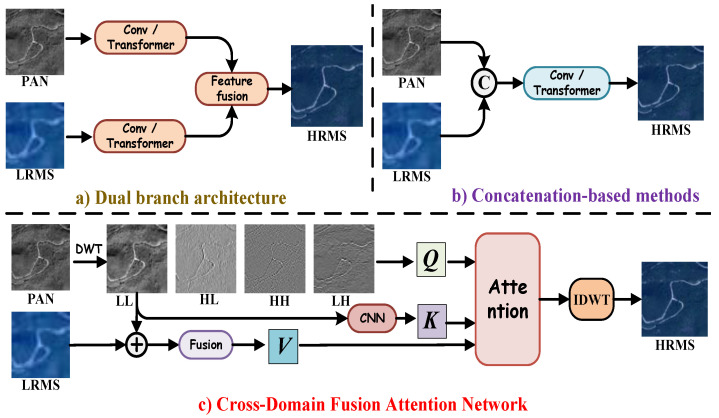
DL-based pansharpening framework. (**a**) Dual-branch architecture. (**b**) Concatenation-based method. (**c**) The proposed Cross-Domain Fusion Attention Network.

Deep neural networks (DNNs), leveraging their nonlinear mapping capabilities and advanced feature extraction mechanisms, have emerged as the predominant approach in the pansharpening domain [25,26]. Within dual-branch architectures (Figure 1a), Zhang et al. [27] employed multiple CNNs to extract complementary features from LRMS and PAN images. By effectively integrating the high spatial resolution of PAN images with the rich spectral information of LRMS images, this approach enhances fusion performance. In contrast, concatenation-based methods (Figure 1b) adopt a different strategy. Ozcelik et al. [28] introduced PCGAN, a generative adversarial network (GAN)-based framework that conceptualizes pansharpening as a colorization process applied to the PAN image. This method enhances spatial fidelity while preserving spectral consistency. To further enhance fusion quality, Lu et al. [29] introduced the multi-scale self-attention network (MSAN), which combines a feature extraction branch with a self-attention mechanism. This approach allows for the dynamic incorporation of detailed spectral information, enhancing the effectiveness of pansharpening. More recently, Quan et al. [30] introduced a dual-parallel Transformer designed to enhance semantic reconstruction, effectively capturing both spatial and spectral dependencies for higher-quality fused remote sensing images. However, despite their advantages, both CNN-based and Transformer-based architectures exhibit a fundamental limitation: their tendency to prioritize low-frequency information. This issue primarily arises due to the direct fusion of spatial features, which fails to adequately disentangle high-frequency textures from background structures. As a result, generated images often suffer from blurred textures and a loss of fine-grained details, compromising the overall sharpness and structural integrity of the output. Moreover, these methods often fail to explicitly quantify or control the information flow across modalities. Without an information-theoretic constraint or awareness, such models may lead to spectral distortion or loss of high-frequency spatial entropy during fusion.

The wavelet-domain transform [31,32,33] has emerged as a powerful framework for pansharpening, offering significant advantages over spatial-domain fusion methods. By enabling multi-scale image analysis, it effectively mitigates the inherent trade-offs between spectral and spatial information representation. Huang et al. [31] introduced a wavelet-based super-resolution CNN, which operates directly in the wavelet domain, leveraging wavelet coefficients to preserve high-frequency details and enhance spatial resolution. However, CNNs inherently prioritize low-frequency features, making them less effective at preserving high-frequency details [34], which ultimately degrades fusion quality. In contrast, the Wavelet Pyramid Recurrent Structure-Preserving Attention Network [32] adopted a distinct strategy, independently processing low- and high-frequency components to retain structural integrity while refining fine details. However, conventional wavelet transforms combined with Transformers fail to simultaneously capture and integrate information across different frequency bands and the unique spectral channels of remote sensing images. Huang et al. [33] utilized wavelet transform to achieve precise frequency separation, generating frequency queries, spatial keys, and fusion values that are based on the physical significance of the different features, thereby enhancing the capture of targeted frequency-domain information.

Inspired by the need for entropy-aware and information-preserving fusion mechanisms, and building upon recent wavelet-domain advances, we propose a novel Cross-Domain Fusion Attention Network (CDFAN), illustrated in Figure 1c. The architecture comprises two synergistic modules: the Multi-Domain Interactive Attention (MDIA) and the Spatial Multi-Scale Enhancement (SMCE) module. The MDIA module first applies discrete wavelet transform (DWT) to the PAN image, explicitly decomposing it into four frequency sub-bands that serve as wavelet-domain queries. The low-frequency sub-band, which retains the core spatial structure, is separately processed via a Local Multiscale Spatial Feature Extraction (LMSF) module to form spatial-domain keys. Simultaneously, the LRMS image undergoes enhancement through a Channel Feature Enhancement (CFE) module and is further processed by a multi-scale large-kernel convolution (MLK) module to generate multi-domain values. These query–key–value triplets, drawn from both the spatial and wavelet domains, are fused using a cross-domain attention mechanism that enables entropy-aware interaction and frequency-specific representation learning. In parallel, the SMCE module again applies DWT to the PAN image, followed by an Expert Feature Compensator (EFC), which dynamically adjusts the contribution of multi-scale features using adaptive weighting. This module integrates convolutional operations at different scales to robustly capture both global contextual cues and fine-grained textures, enhancing spatial representational capacity. Notably, our work is partly inspired by the SFIIN framework [35], which introduced a dual-branch architecture that fuses spatial and frequency information using Fourier decomposition. However, our method diverges fundamentally by operating in the wavelet domain and incorporating a cross-domain attention mechanism, rather than relying on global Fourier representations and conventional convolution-based fusion. The explicit construction of wavelet-domain queries and spatial-domain keys, along with adaptive multi-scale enhancement through EFC, distinguishes our approach and enhances its ability to capture localized frequency textures while maintaining entropy structure.

Our contributions can be summarized as follows.

1. We introduce a novel MDIA mechanism that leverages DWT to achieve explicit frequency separation. By processing frequency sub-bands as wavelet-domain queries and integrating spatial-domain keys and multi-domain values, the model effectively captures frequency-specific information, leading to enhanced spatial–spectral representation.
2. We propose the EFC branch, which employs an adaptive feature weighting mechanism to dynamically balance global contextual awareness and fine-grained spatial detail preservation. By integrating multi-scale convolutional operations, the EFC enhances hierarchical spatial feature fusion, improving the model’s ability to capture fine-grained structures in high-resolution remote sensing images.
3. Experiments conducted on three distinct pansharpening datasets showcase the superiority of our approach compared to state-of-the-art (SOTA) methods, resulting in improved visual quality and superior quantitative metrics.

## 2. Related Work

### 2.1. Wavelet Transform of Images

Wavelet decomposition is a powerful method used in image processing to break down an image into various frequency components, distinguishing between coarse and fine details [36,37]. By applying wavelet transforms, an image is separated into a low-frequency sub-band that maintains the overall structure, and three high-frequency sub-bands that capture more intricate patterns. This method involves the use of four distinct filters, namely, ${\mathrm{LL}}^{T}$, ${\mathrm{LH}}^{T}$, ${\mathrm{HL}}^{T}$, and ${\mathrm{HH}}^{T}$, each serving to extract specific frequency components.

The filter pairs used in the wavelet decomposition are defined as follows:

$$L=\frac{1}{\sqrt{2}}\left[\begin{array}{cc} 1 & 1 \end{array}\right],\;H=\frac{1}{\sqrt{2}}\left[\begin{array}{cc} -1 & 1 \end{array}\right]$$

These filters are then applied to the image, X, with a stride of 2. This operation results in the following sub-bands: (1) Low-frequency approximation sub-band: $X_{LL}$. (2) High-frequency sub-bands: $X_{LH}$ (vertical details), $X_{HL}$ (horizontal details), and $X_{HH}$ (diagonal details). Thus, the wavelet transform decomposes the image X into four sub-bands, where the resolution of the original image is effectively halved in the process. The wavelet transform is reversible and lossless due to the orthogonality of the filters L and H. As a result, the original image can be accurately reconstructed from these four sub-bands. The operations for decomposition and reconstruction are represented as

(1) $$X_{LL},X_{LH},X_{HL},X_{HH}=\mathrm{IDWT}\left(X\right)$$

(2) $$\mathrm{IDWT}(X_{LL},X_{LH},X_{HL},X_{HH})=X$$

where DWT denotes the discrete wavelet transform (DWT) and IDWT represents the inverse discrete wavelet transform (IDWT).

Unlike the Fourier transform, which operates solely in the frequency domain, wavelet decomposition offers a multiresolution analysis. This allows for both spatial- and frequency-domain features of an image to be captured simultaneously. Moreover, the wavelet domain provides a compact and information-preserving representation of image content, facilitating localized entropy analysis and efficient separation of high-frequency details, which are often rich in structural and textural information. This capacity to retain localized information content with minimal redundancy makes wavelet transform particularly suitable for entropy-aware deep learning applications, where fine-grained spatial patterns and frequency structures are critical.

### 2.2. CNN-Based Pansharpening

Convolutional neural networks (CNNs) have been widely adopted in pansharpening due to their strong representation capability and end-to-end learning nature.

He et al. [38] proposed dynamic pansharpening CNNs (DyPNNs) that generate spatially adaptive fusion rules via a learn-to-learn framework, achieving improved spatial and spectral performance across multiple hyperspectral datasets. Girish et al. [39] proposed a Transformer-based Adaptive 3D Residual CNN (TA-3DRCNN) for pansharpening, combining MS and PAN images via sparse representation and deep learning to enhance spatial resolution while minimizing distortions. Wang et al. [40] proposed PSCINN, a multi-scale conditional invertible neural network for pansharpening, which preserves spectral fidelity and texture by leveraging a latent representation guided by the PAN image. A specially designed invertible block enables accurate spectral recovery, leading to superior performance over existing methods. Fang et al. [41] proposed SDRCNN, a lightweight single-branch convolutional neural network for pansharpening, featuring a dense residual structure to balance accuracy and efficiency.

Despite their success, CNN-based methods are fundamentally limited by their local receptive fields and convolutional inductive biases, which restrict their ability to model long-range dependencies and global contextual relationships. Moreover, most of these networks treat PAN and LRMS inputs independently, without explicitly modeling the mutual information between spectral and spatial domains. These limitations have motivated the development of Transformer-based architectures, which aim to overcome these constraints through global attention and multiresolution analysis.

### 2.3. Transformer-Based Pansharpening

Transformers, initially popularized in natural language processing (NLP), have been increasingly adopted in computer vision due to their strong capability to capture long-range dependencies—an advantage over CNNs with inherently limited receptive fields. This property makes Transformers particularly suitable for pansharpening tasks. Su et al. [42] proposed a Transformer-based regression network that effectively captures global spectral and spatial details. Zhang et al. [43] integrated CNNs with Transformers to enhance shared feature extraction while mitigating redundancy. Bandara et al. [44] introduced HyperTransformer, which combines CNNs and Transformers with a multi-scale fusion strategy to improve fusion quality. Guan and Lam [45] developed MDANet, a multistage dual-attention fusion network that extracts and integrates key features to reconstruct latent HRMS images. Li et al. [46] presented a Transformer-based deep unfolding network (DUN), incorporating a local–global module designed as an image denoiser, effectively capturing dependencies across both local regions and global contexts—addressing challenges associated with varying object scales and spatial distributions in remote sensing imagery. To reduce computational complexity while retaining performance, Hou et al. [47] proposed a lightweight pansharpening framework based on a first-order linearly evolved Transformer, replacing conventional cascaded Transformers with a 1D linear convolutional chain. Most recently, Ye et al. [48] introduced a multi-scale hybrid spatial–spectral Transformer (MSHST) built upon a multi-scale convolutional sparse coding (MS-CSC) observation model. Their approach captures global spatial dependencies and inter-band spectral correlations, achieving state-of-the-art performance in both reduced-resolution and full-resolution evaluations.

Despite these advancements, existing approaches often overlook the underlying entropy structure across spectral and frequency channels. Most networks fail to explicitly model or preserve mutual information between PAN and LRMS inputs, leading to either spectral distortion or redundant feature learning. Therefore, integrating wavelet-based frequency decomposition with attention mechanisms, particularly within Transformer architectures, offers a promising direction to build entropy-aware and information-efficient pansharpening models.

## 3. Proposed Method

In this section, we provide a comprehensive explanation of the architecture of the Cross-Domain Fusion Attention Network (CDFAN), which comprises two pivotal modules: the Multi-Domain Interactive Attention (MDIA) and the Spatial Multi-Scale Enhancement module (SMCE).

### 3.1. Overview of Network Framework

**Figure 2:**
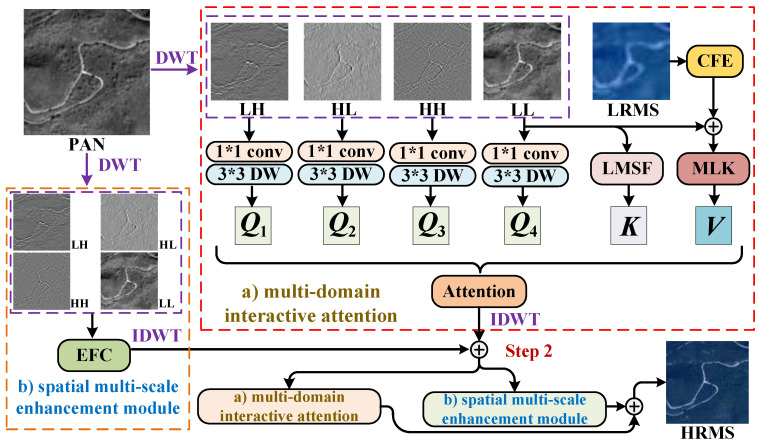
The overall workflow of our CDFAN.

Figure 2 provides an overview of the proposed CDFAN framework, which is constructed through two progressive stages. Given the LRMS image $Y\in R^{h\times w\times S}$ and the PAN image $P\in R^{H\times W\times 1}$, both capturing the same scene, we first upsample the LRMS image using bilateral interpolation. Then, we apply a feature extraction module consisting of a 5 × 5 2D convolution, ReLU activation, and a 3 × 3 2D convolution to map the PAN and upsampled LRMS images into a unified spectral feature space, extracting shallow features, where the spectral dimension is set to 32 in this study. This process can be represented as

(3) $$Y_{s}=\mathrm{In}\_\mathrm{head}\left(Y\right),\;P_{s}=\mathrm{In}\_\mathrm{head}\left(\mathrm{upsample}\left(P\right)\right)$$

where upsample is the upsample operation, In_head is the shallow feature extraction module, $Y_{s}\in R^{H\times W\times C}$ and $P_{s}\in R^{H\times W\times C}$ is the shallow feature, and *C* is the number of channels. Then, we utilize the MDIA and SMCE separately to extract global and local information in a decoupled manner, ensuring entropy-aware feature learning and minimizing cross-domain information loss during fusion.

In the MDIA, we first apply wavelet transform to the feature $Y_{s}$ to derive sub-bands:

(4) $$P_{LL},P_{LH},P_{HL},P_{HH}=\mathrm{DWT}\left(P_{s}\right)$$

We first extract the spatial key from the separated low-frequency feature $P_{LL}\in R^{\frac{H}{2}\times \frac{W}{2}\times C}$ using the Local Multiscale Spatial Feature Extraction (LMSF) module. Then, our multi-scale large-kernel convolution interaction module (MLK) aggregates these low-frequency features with the Channel Feature Enhancement (CFE) module $Y_{s}$ to generate a multi-domain value. Next, we apply convolution to the four separated frequency features to obtain the wavelet-domain query. This wavelet-domain query, together with the spatial-domain key and the multi-domain value, is used to construct a multi-domain global information attention mechanism. The mathematical formulation of this global structure modeling process is as follows:

(5) $$F_{global}=\mathrm{Attention}\left(Y_{s},P_{s}\right)=\left(\underset{wavelet-domain\;query}{\underset{︸}{\left(P_{LL},P_{LH},P_{HL},P_{HH}\right)}},\underset{spatial\;key}{\underset{︸}{\mathrm{LMSF}(P_{LL})}},\underset{multi-domain\;value}{\underset{︸}{\mathrm{MLK}(P_{LL}+\mathrm{CFE}(Y_{s}))}}\right)$$

In the SMCE, we further process the four frequency-domain features extracted via DWT using an Expert Feature Compensator (EFC) mechanism. This approach integrates information at different local spatial scales to enhance feature representation. This process is illustrated below:

(6) $$F_{local}=\mathrm{EFC}\left(P_{LL},P_{LH},P_{HL},P_{HH}\right)$$

After extracting the local features $F_{local}\in R^{\frac{H}{2}\times \frac{W}{2}\times C}$ and global features $F_{global}\in R^{\frac{H}{2}\times \frac{W}{2}\times C}$ from MDIA and SMCE, respectively, we first obtain a preliminary reconstructed image via inverse discrete wavelet transform (IDWT). These features are then integrated through element-wise addition to produce the fused image $F_{fusion}\in R^{H\times W\times C}$, enabling collaborative information exchange and complementary feature fusion. This operation is mathematically expressed as

(7) $$F_{fusion}=\mathrm{IDWT}\left(F_{global}\right)+\mathrm{IDWT}\left(F_{local}\right)$$

The above process completes step 1. In step 2, within the global information modeling, $F_{fusion}$ replaces $Y_{s}$ from Equation (7) to construct a more enriched and comprehensive multi-domain feature representation. The corresponding mathematical formulation is as follows:

(8) $${\bar{F}}_{global}=\left(\underset{wavelet-domain\;query}{\underset{︸}{\left(P_{LL},P_{LH},P_{HL},P_{HH}\right)}},\underset{spatial\;key}{\underset{︸}{\mathrm{LMSF}(P_{LL})}},\underset{multi-domain\;value}{\underset{︸}{\mathrm{MLK}(P_{LL}+\mathrm{CFE}(F_{\mathrm{fusion}}))}}\right)$$

The information aggregation method for $F_{local}$ remains consistent with Equation (8). Thus, the final form of the fused output feature is given by $F_{fusion}=\mathrm{IDWT}\left({\bar{F}}_{global}\right)+\mathrm{IDWT}\left(F_{local}\right)$.

### 3.2. Multi-Domain Interactive Attention

#### 3.2.1. Wavelet-Based Query Generation

**Figure 3:**
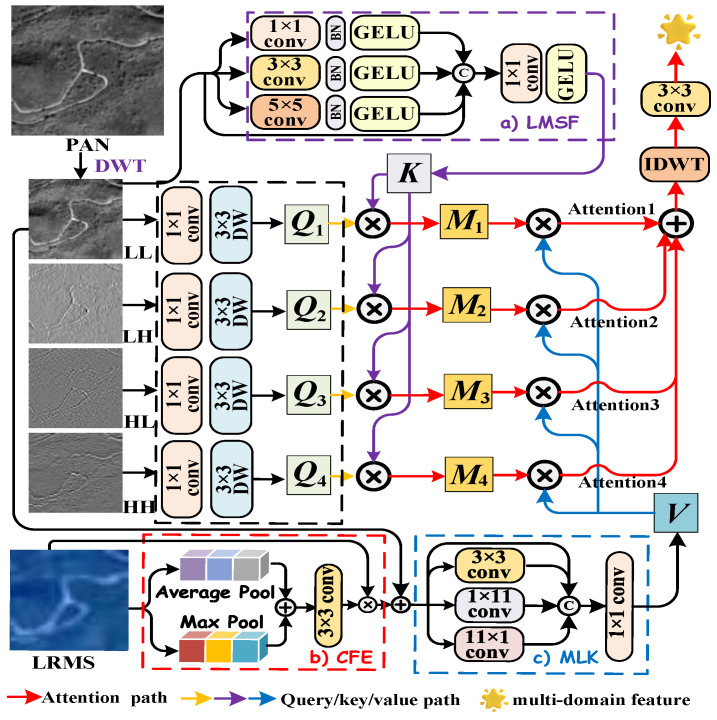
Flowchart of the proposed Multi-Domain Interactive Attention framework. (**a**) Details of Local Multiscale Spatial Feature Extraction (LMSF). (**b**) Structure of the Channel Feature Enhancement (CFE) module. (**c**) Structure of the multi-scale large-kernel convolution interaction module (MLK).

It is well established that the discrete wavelet transform (DWT) decomposes an image into low-frequency features and high-frequency components. The low-frequency features capture the general spatial structure of the image, whereas the high-frequency components encode edge details and fine textures, both of which play a vital role in feature representation. Inspired by [33], we employ a feature extraction module composed of a 3 × 3 2D convolution and a depthwise (DW) convolution to treat all four decomposed components as active information probes, serving as the queries in an attention framework. This enables dynamic filtering and aggregation of relevant information, promoting frequency-aware mutual information transfer between the PAN and LRMS modalities, and enhancing cross-domain consistency. The process, highlighted by the black dashed box in Figure 3, is mathematically formulated as follows:

(9) $$\begin{array}{cc} \; & Q_{LL}={\mathrm{DW}}_{3\times 3}\left({\mathrm{Con}}_{3\times 3}\left(P_{LL}\right)\right),\\ \; & Q_{LH}={\mathrm{DW}}_{3\times 3}\left({\mathrm{Con}}_{3\times 3}\left(P_{LH}\right)\right),\\ \; & Q_{HL}={\mathrm{DW}}_{3\times 3}\left({\mathrm{Con}}_{3\times 3}\left(P_{HL}\right)\right),\\ \; & Q_{HH}={\mathrm{DW}}_{3\times 3}\left({\mathrm{Con}}_{3\times 3}\left(P_{HH}\right)\right), \end{array}$$

where DW represents the depthwise convolution and Conv is the convolution.

#### 3.2.2. Spatial-Domain Key Representation

The key K serves as a retrievable candidate identifier for the query Q, necessitating precise and comprehensive information matching. In this work, we utilize the low-frequency feature $P_{LL}$, which encapsulates the overall spatial structure, as the background representation. To enhance multi-scale spatial modeling while preserving local spatial structures, we propose the Local Multiscale Spatial Feature Extraction (LMSF) module. The LMSF module utilizes a 3×3 convolution to capture spatial correlations from neighboring pixels, a 5×5 average pooling to extract medium-scale rotation-invariant features, and a 1×1 convolution to refine pixel-level details. As illustrated by the purple dashed box in Figure 3, the multi-scale feature extraction process can be mathematically expressed as follows:

(10) $$\begin{array}{cc} \; & F_{1}^{spa}=\mathrm{GELU}\left(\mathrm{BN}\left({\mathrm{Con}}_{3\times 3}\left(P_{LL}\right)\right)\right),\\ \; & \;F_{2}^{spa}=\mathrm{GELU}\left(\mathrm{BN}\left({\mathrm{Avgpool}}_{5\times 5}\left(P_{LL}\right)\right)\right),\\ \; & F_{3}^{spa}=\mathrm{GELU}\left(\mathrm{BN}\left({\mathrm{Con}}_{1\times 1}\left(P_{LL}\right)\right)\right), \end{array}$$

where BN is the batch normalization and GELU denotes Gaussian error linear unit. Here, $F_{1}^{spa}$, $F_{2}^{spa}$, and $F_{3}^{spa}$ share the same spatial dimensions as $F_{s}$. These multi-scale features are concatenated with the original input before undergoing a 1×1 convolution, enabling dynamic spatial information integration for each pixel. The final spatial-domain key is given by

(11) $$K=\mathrm{GELU}({\mathrm{Con}}_{1\times 1}\left(\mathrm{Cat}\left(F_{1}^{spa},F_{2}^{spa},F_{3}^{spa},P_{LL}\right)\right))$$

#### 3.2.3. Multi-Domain Value Generation

In the attention mechanism, V represents the actual information that is transmitted and aggregated to construct the final context vector. Therefore, the information contained in V should be rich, multi-dimensional, and capable of capturing high-level semantic representations. Building upon this principle, we propose integrating the spectral information from LRMS images with the spatial information from PAN images to facilitate feature interaction and enhancement. Specifically, we design a Channel Feature Enhancement (CFE) module, which operates on the shallow features of the upsampled LRMS. This attention mechanism enables the model to selectively amplify informative spectral features while suppressing anomalous variations, thereby improving the quality of the fused representation. The CFE module first applies both adaptive max pooling and average pooling to the input feature maps, aiming to extract complementary feature representations at different scales. The outputs from these two pooling operations are then aggregated via element-wise summation and subsequently processed through a 3×3 convolution followed by a sigmoid activation function, generating a channel attention map. Finally, the original input feature maps are modulated by this attention map through element-wise multiplication, producing a refined representation with enhanced spectral and spatial features. The CFE function is illustrated by the red dashed box in Figure 3, and its detailed formulation is expressed mathematically as follows:

(12) $$F_{CFE}=\sigma \left({\mathrm{Conv}}_{3\times 3}\left(\mathrm{MaxPool}\left(Y_{s}\right)+\mathrm{Avgpool}\left(Y_{s}\right)\right)\right)$$

(13) $${\bar{F}}_{CFE}=F_{CFE}⊙Y_{s}$$

where ⊙ represents element-wise multiplication, σ denotes the sigmoid activation function, while MaxPool and AvgPool represent the max pooling and average pooling operations, respectively.

After obtaining the channel-enhanced features of LRMS, we further introduce the multi-scale large-kernel convolution interaction module (MLK) to jointly model the low-frequency features extracted from both the LRMS and PAN images, thereby enhancing the complementarity between multi-modal information. The MLK module employs a multi-scale convolutional strategy that integrates different types of convolutional kernels to capture spatial information at varying scales. Specifically, a compact square convolutional kernel using a 3×3 convolution extracts local detailed features, while two orthogonal band kernels with 1×11 and 11×1 convolutions enhance cross-directional information fusion. This multi-scale structure not only strengthens local feature representation but also effectively mitigates content sparsity and facilitates deep cross-modal feature fusion, ultimately improving the overall feature representation. Mathematically, for fusion feature $F_{f}=P_{LL}+{\bar{F}}_{CFE}$, the MLK module, highlighted by the blue dashed box in Figure 3, can be formulated as

(14) $$\begin{array}{cc} \; & F_{f1},F_{f2},F_{f3},F_{f4}=\mathrm{Split}\left(F_{f}\right),\\ \; & F_{f1}^{\prime }={\mathrm{DW}}_{3\times 3}\left(F_{f1}\right),\\ \; & F_{f2}^{\prime }={\mathrm{DW}}_{1\times 11}\left(F_{f2}\right),\\ \; & F_{f3}^{\prime }={\mathrm{DW}}_{11\times 1}\left(F_{f3}\right),\\ \; & F_{f4}^{\prime }=F_{f4}, \end{array}$$

Finally, the outputs from each branch are concatenated:

(15) $$V=\mathrm{GELU}({\mathrm{Con}}_{1\times 1}\left(\mathrm{Cat}\left(F_{f1}^{\prime },F_{f2}^{\prime },F_{f3}^{\prime },F_{f4}^{\prime }\right)\right)$$

#### 3.2.4. Multi-Domain Attention Product

After obtaining the attention triplet representations from different domains, we further employ an attention-based mechanism to adaptively aggregate cross-domain information and enhance the representation of fused features. First, we compute four independent frequency-domain attention maps ($M_{i}$) between the wavelet-domain query features and the spatial-domain key feature, aiming to capture the interdependency between different frequency characteristics and the global spatial structure. Subsequently, the dual-domain attention map is multiplied by the multi-domain value to realize the multi-domain and multi-scale wavelet attention network. This process enables deep feature fusion and joint representation across the three domains, thereby enhancing the robustness, discriminative power, and expressiveness of the learned features. The overall framework, which is illustrated in Figure 3, is illustrated as follows:

(16) $$\begin{array}{cc} \; & M_{1}=\mathrm{softmax}\left(Q_{LL}⊗K\right),M_{2}=\mathrm{softmax}\left(Q_{LH}⊗K\right),\\ \; & M_{3}=\mathrm{softmax}\left(Q_{HL}⊗K\right),M_{4}=\mathrm{softmax}\left(Q_{HH}⊗K\right),\\ \; & {\mathrm{Attention}}_{1}=M_{1}⊗V,{\mathrm{Attention}}_{2}=M_{2}⊗V,\\ \; & {\mathrm{Attention}}_{3}=M_{3}⊗V,{\mathrm{Attention}}_{4}=M_{4}⊗V, \end{array}$$

Finally, we leverage the lossless reconstruction property of the inverse discrete wavelet transform (IDWT) to perform an inverse transformation on the four independent multi-domain attention maps, effectively integrating information across different frequencies and spatial domains. Subsequently, we apply element-wise summation followed by a 3×3 convolution to further enhance multi-domain feature fusion, resulting in the initially reconstructed image. The detailed process is illustrated as follows:

(17) $$F={\mathrm{Con}}_{3\times 3}\left(\mathrm{IDWT}\left({\mathrm{Attention}}_{1}+{\mathrm{Attention}}_{2}+{\mathrm{Attention}}_{3}+{\mathrm{Attention}}_{4}\right)\right)$$

Although the DWT provides explicit frequency separation and compact representation, it may introduce local artifacts or cause attenuation of subtle spatial structures, especially in high-frequency bands. To address this, the proposed MDIA module constructs an attention mechanism that jointly leverages wavelet-domain queries and spatial-domain keys. This design enables dynamic alignment of frequency-aware features with spatial structures preserved in the low-frequency components. By computing attention weights across multi-domain representations, the model adaptively restores fine details and alleviates the local distortions introduced by DWT.

### 3.3. Spatial Multi-Scale Enhancement Module

PAN images contain rich spatial details and structural information, which are crucial for restoring high-resolution spatial features in HRMS images. However, spatial feature extraction is highly sensitive to variations in the receptive field of convolutional kernels. Larger convolutional kernels capture broader spatial regions, enabling the extraction of global structures and long-range dependencies, whereas smaller convolutional kernels focus on fine-grained textures and local details. To mitigate the impact of receptive field sensitivity, we propose an Expert Feature Compensator (EFC), which operates on four decomposed frequency-domain representations to construct a Spatial Multi-Scale Enhancement (SMCE) module branch. The EFC dynamically regulates the contributions of multi-scale features through an adaptive feature weighting mechanism, ensuring an optimal balance between global contextual information and fine-grained spatial details, while preserving essential information content and maximizing local spatial entropy. By integrating multi-scale convolutional operations, it effectively fuses hierarchical spatial features within PAN images, thereby enhancing spatial information integrity and representation capability.

**Figure 4:**
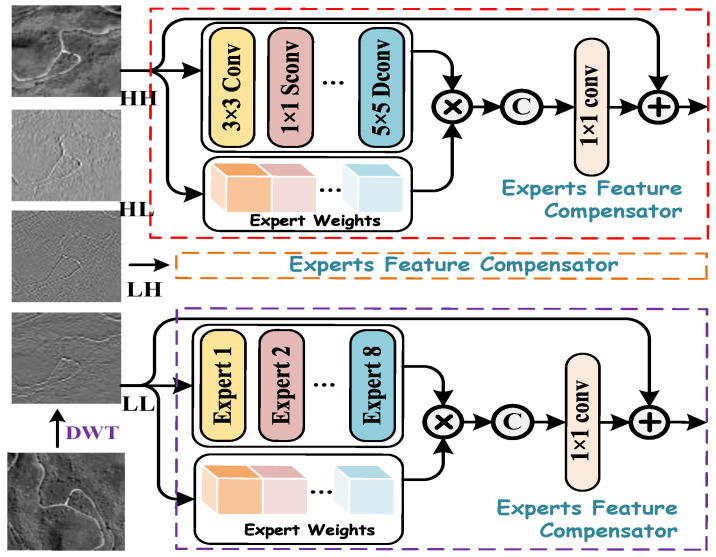
Illustration of the Spatial Multi-Scale Enhancement module.

Building on the foundational design principles of efficient CNN architectures, we carefully select a set of sparse CNN operations to construct parallel layers, collectively referred to as experts. These experts are carefully selected to provide diverse feature representations, including average pooling with a 3 × 3 receptive field to capture global information; separable convolutions with kernel sizes of 1×1, 3×3, 5×5, and 7×7 for multi-scale feature extraction; and dilated convolutions with kernel sizes of 3×3 and 5×5 to expand the receptive field without increasing the number of parameters. Each of these experts processes the input independently, and their outputs are later fused using a self-attention-based selection mechanism. Figure 4 illustrates the EFC framework, highlighting the interaction between expert weights, convolutional experts, and the final feature fusion process. Similarly, we first apply DWT to decompose the PAN image into four frequency sub-images. Each sub-image is then fed into the EFC module to adaptively enhance frequency-specific features and optimize cross-scale information fusion.

Given any frequency-domain separation feature $X_{l-1}\in R^{H\times W\times C}$, we first apply channel-wise global averaging to derive a C-dimensional descriptor $z_{c}\in R^{C}$. The descriptor is computed as follows:

(18) $$z_{c}=\frac{1}{N}\sum_{(i,j)\in \Omega }X_{l-1}\left(i,j\right),$$

where N=H×W is the total number of spatial locations, and Ω represents the set of all spatial coordinates (i,j) within the feature map. The computed descriptor vector $z_{c}$ is then transformed via two learnable projection matrices to obtain the expert weight vector:

(19) $$T_{l}=W_{2}\cdot \mathrm{ReLU}\left(W_{1}z_{c}\right),$$

Here, the coefficient vector of each expert is allocated corresponding to the learnable weight matrices $W_{1}\in R^{T\times C}$ and $W_{2}\in R^{O\times T}$, where *O* and *T* represent the number of expert operations and the feature transformation dimension, respectively. The resulting vector $T_{l}$ encodes the relative importance of each expert and is subsequently used to weight the expert outputs. Moreover, to maintain spatial consistency, we apply zero-padding to the input feature maps processed by each expert. The final aggregated output of the *l*-th MEFC module is then computed as

(20) $$X_{l}={\mathrm{Con}}_{1\times 1}\left(\sum_{o=1}^{O}f_{\exp}\left(X_{l-1},T_{l}^{\left(o\right)}\right)\right)+X_{l-1},$$

where $f_{\exp}\left(\cdot \right)$ denotes the expert-specific feature transformation. The four frequency-domain representations processed by the EFC are first concatenated and then refined using a 3 × 3 convolution. Subsequently, an IDWT is applied to effectively explore spatial information across different frequency domains, enhancing the model’s robustness in handling complex textured remote sensing images.

## 4. Experiments and Evaluation

**Figure 5:**
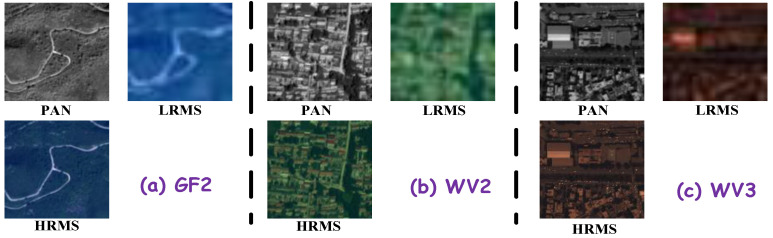
MS and PAN image samples used in experiments.

This section outlines the experimental framework used to assess the efficacy of the proposed CDFAN method, leveraging remote sensing imagery from three satellite platforms: WorldView-3 (WV-3), WorldView-2 (WV-2), and GaoFen-2 (GF-2). Figure 5 presents the MS and PAN images for use in the experiment. To simulate realistic low-resolution data, we apply downsampling techniques to the original high-resolution imagery, as prescribed by Wald et al. [49]. This downsampling ensures a comprehensive evaluation of the fusion model’s ability to restore information from low-resolution inputs. The three datasets are further split into non-overlapping subsets for training and testing, and the detailed properties of these datasets are summarized in Table 1.

### 4.1. Quality Metrics and Cutting-Edge Competitors

To thoroughly assess the quantitative performance of all the competing approaches, we utilize six well-established evaluation metrics, peak signal-to-noise ratio (PSNR), structural similarity (SSIM), spectral angle mapper (SAM), and erreur relative globale admensionnelle de synthese (ERGSA) [50], along with universal image quality indices for 4-band (Q4) and 8-band (Q8) images [51]. In addition to traditional quality metrics, we incorporate entropy-aware indicators such as the QNR [52], $D_{s}$ [53], and $D_{\lambda }$ [54] to assess the degree of information distortion across the spatial and spectral domains, especially in the absence of ground-truth reference images. These metrics serve as information-theoretic tools to quantify mutual information preservation and redundancy reduction.

The comparative study involves three model-based techniques—GSA [55], SFIM [56], and [57]—alongside eight advanced deep learning-based methods: PanFormer [58], CTINN [59], LightNet [60], SFIIN [35], MutInf [61], MDCUN [62], LGTEUN [46], SSDBPN [63], BiMPan [64], and PAPS [65].

### 4.2. Experimental Setup

To guarantee uniformity and replicability across all experiments, we performed our computations using Python 3.7 alongside PyTorch 1.9.0, leveraging a computational platform equipped with NVIDIA GeForce RTX 3090 GPUs. For the deep learning-based pansharpening approach, the training configuration was established as follows: a batch size of 4 was adopted, with the GF-2 and WV-2 datasets undergoing 500 training epochs, while the WV-3 dataset was trained for 140 epochs. The starting learning rate was configured at $1.5\times 10^{-3}$, subject to a decay factor of 0.85 applied at intervals of 100 epochs.

### 4.3. Synthetic Data Experiments

In this section, we present the outcomes generated by all the evaluated methods across the three chosen datasets, namely, the WV-3, WV-2, and GF-2 datasets. The optimal values are marked in red, while the second-best values are indicated in blue.

#### 4.3.1. Quantitative Analysis

The CDFAN approach demonstrates exceptional performance across all major evaluation metrics on the WorldView-3 dataset, as summarized in Table 2. It achieves a notable PSNR of 32.5080, surpassing the second-place method, BiMPan, by 0.2230, highlighting CDFAN’s superior capacity for preserving critical information content and reducing cross-domain redundancy, ultimately leading to higher image quality. Additionally, CDFAN shows a marked improvement in the ERGAS metric, registering a value of 2.5389, which is 21.36% better than the PAPS method (2.6127). This emphasizes the effectiveness of SCTA not only in preserving image fidelity but also in providing a more accurate and computationally efficient reconstruction, making it particularly suitable for high-precision tasks that require optimization.

On the WorldView-2 dataset, CDFAN consistently outperforms competing methods, as evidenced by the results in Table 3. It secures the highest PSNR of 42.7719, exceeding LGTEUN by 0.1475 and PAPS by 0.1403, which underscores CDFAN’s superior ability to preserve image fidelity. Furthermore, CDFAN leads the SSIM metric with a score of 0.9789, indicating that it consistently maintains a high degree of structural similarity in the reconstructed images. CDFAN also excels in the SAM metric with a notably low score of 1.1747, improving by 16.87% over BiMPan (1.2433), thus demonstrating its proficiency in preserving angular fidelity and minimizing distortion in pixel vectors.

On the GaoFen-2 dataset, CDFAN proves to be the dominant method, leading almost every evaluation metric, as detailed in Table 4. It achieves the highest PSNR score of 46.8108, outperforming the second-best LGTEUN by a significant 0.9744, indicating its exceptional capability to maximize information fidelity and maintain entropy consistency, producing high-quality and perceptually faithful reconstructions. In terms of SSIM, CDFAN maintains its dominance with a score of 0.9868, outperforming BiMPan (0.9822) by 0.0046, further confirming its ability to preserve image structure. Additionally, CDFAN leads the Q4 metric with a value of 0.9117, surpassing BiMPan (0.8928) by 0.0189, thus highlighting its superior performance in image quantization and quality evaluation.

In conclusion, CDFAN stands out as the clear leader across all datasets, consistently demonstrating its exceptional ability to preserve image quality, structural integrity, and computational efficiency. With top scores in PSNR, SSIM, and ERGAS on the WorldView-3 and WorldView-2 datasets, and leading results in PSNR and SSIM on GaoFen-2, CDFAN proves to be the most reliable and effective method for remote sensing image reconstruction, offering both high-quality results and computational efficiency across a wide range of benchmark datasets.

#### 4.3.2. Qualitative Analysis

**Figure 6:**
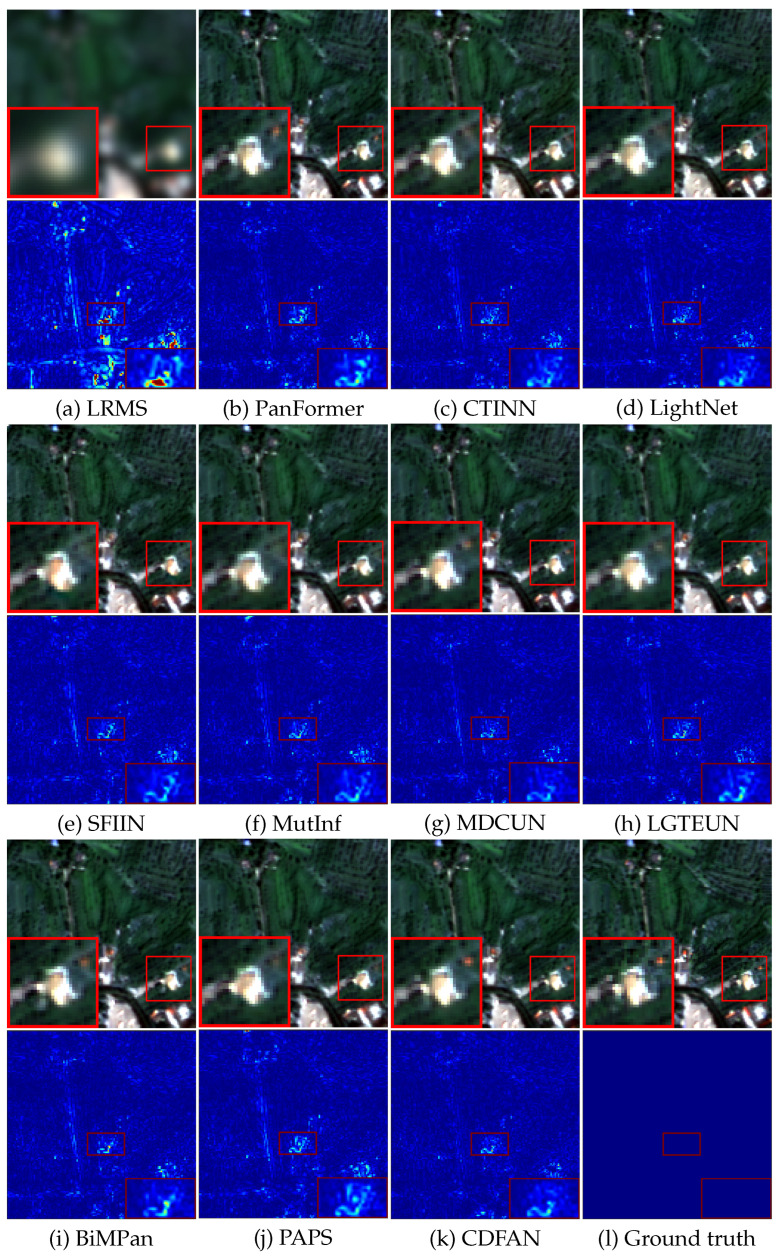
Visual comparisons on typical GF-2 data. (**a**) Observed data. (**b**–**k**) The results from GSA, SFIM, Wavelet, PanFormer, CTINN, LightNet, SFIIN, MutInf, MDCUN, LGTEUN, BiMPan, PAPS, and CDFAN, respectively. (**l**) Ground truth.

For visual evaluation, Figure 6 presents representative examples and corresponding error maps recovered from the GF-2 dataset. As observed in the figure, all models yield relatively satisfactory results. However, upon closer inspection of the zoomed-in thumbnails, it becomes apparent that nearly all the comparative methods struggle to effectively reconstruct the rooftop area in the upper-right corner, particularly failing to recover the original red pseudo-color. While BiMPan and MDCUN partially restore the red rooftop, their results remain dimmer. In contrast, CDFAN delivers clearer results with fewer artifacts, especially in the zoomed-in region, where it more accurately recovers the red rooftop. The error maps, particularly in the magnified areas, reveal that our method tends to produce smoother, more blue-toned results, in contrast to competing methods that integrate HRMS with noticeable defects and noise. Overall, the experimental results demonstrate that our method surpasses other holographic reconstruction approaches. Moreover, the smoother error maps observed in CDFAN’s outputs reflect its capacity to minimize information distortion and preserve entropy structure across spatial scales, leading to better texture fidelity and detail preservation. This superiority is mainly attributed to the comprehensive extraction of both local-global and deeper features enabled by the MDIA and SMCE modules, which significantly enhance the final reconstruction quality.

**Figure 7:**
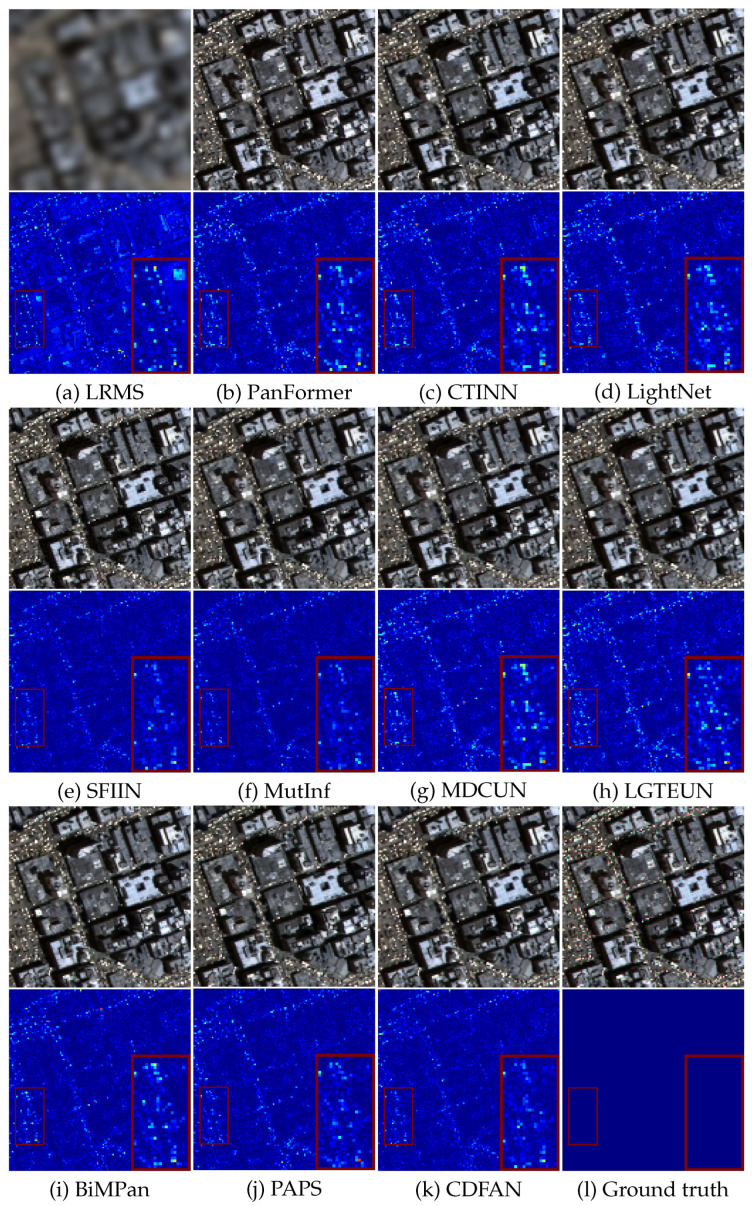
Visual comparisons on typical WV-3 data. (**a**) Observed data. (**b**–**k**) The results from GSA, SFIM, Wavelet, PanFormer, CTINN, LightNet, SFIIN, MutInf, MDCUN, LGTEUN, BiMPan, PAPS, and CDFAN, respectively. (**l**) Ground truth.

For the WV-3 dataset, as shown in Figure 7, it is evident that model-based techniques fail to perform adequately, as their generated results are plagued by abnormal pixels and excessive distortion. On the other hand, all deep learning-based competitors can roughly capture spatial details from the PAN image. However, while other algorithms struggle to restore the bright rooftop colors clearly, the proposed method achieves the most accurate restoration. The error maps, particularly in the magnified regions, indicate that our method tends to generate smoother and more blue-toned results, highlighting the flaws of other methods that fuse HRMSs with more defects and scattered points. In general, the experimental results confirm the superiority of our method over other pansharpening approaches. This advantage arises from the comprehensive extraction of both local-global and deeper features facilitated by the CDFAN and MAC modules, which contribute to the refined quality of the final outcomes. Moreover, visual inspections across multiple samples reveal no noticeable artifacts introduced by the wavelet decomposition, indicating that our MDIA design effectively mitigates potential side effects of DWT.

### 4.4. Effectiveness of CDFAN in Mitigating Entropy Degradation

**Figure 8:**
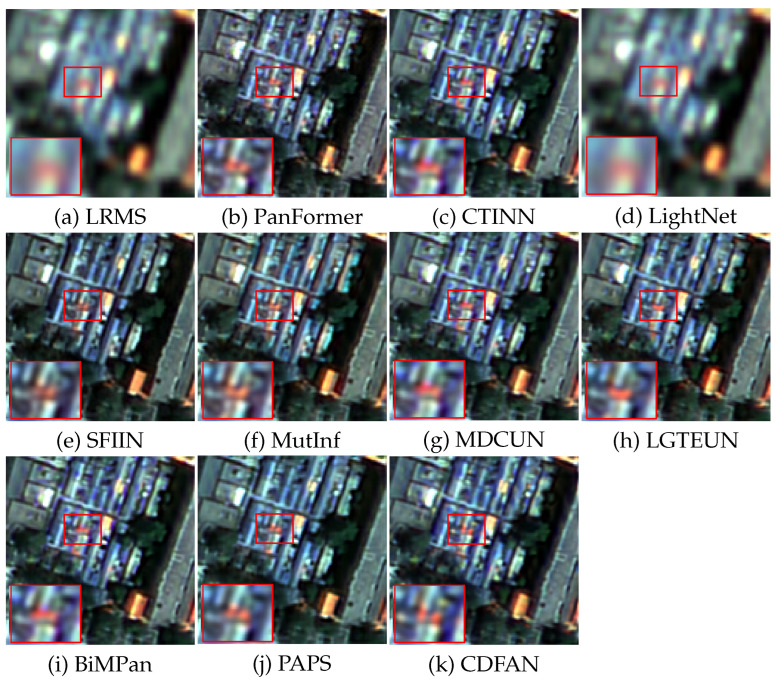
Visual comparisons on typical GF-2 data at full resolution.

To further evaluate the entropy-preserving capability of CDFAN under full-resolution settings, we report three information-theoretic metrics—$D_{\lambda }$, $D_{s}$, and QNR—in Table 5. These metrics quantify the spectral distortion, spatial distortion, and overall fusion quality, respectively, without requiring reference ground-truth HRMS images. In the absence of reference images, we process the full-resolution input directly through a model that was trained on downsampled datasets, producing pansharpened outputs. The PAN images used have dimensions of 512×512, while the LRMS images are 128×128. On the WorldView-3 dataset in Table 5, CDFAN achieves the highest QNR score (0.9539) and the second-best scores in both $D_{\lambda }$ (0.0289) and $D_{s}$ (0.0293), indicating its strong ability to preserve entropy structure during fusion. On the GaoFen-2 dataset, CDFAN outperforms all competitors with $D_{s}=0.1409$ and matches the best QNR score (0.8121) alongside BiMPan. The low values of $D_{\lambda }$ and $D_{s}$ suggest that the proposed network maintains a favorable balance between spatial and spectral fidelity, while its high QNR reflects the model’s superior joint fusion performance. Notably, although BiMPan also achieves competitive QNR, it suffers from higher spectral distortion on WorldView-3 ($D_{s}=0.0305$) and spatial degradation on GaoFen-2 ($D_{s}=0.1419$). In contrast, CDFAN demonstrates more consistent performance across datasets and domains, reinforcing its advantage as an entropy-aware fusion framework capable of minimizing information loss and redundancy. Figure 8 presents a visual comparison of the full-resolution outputs, using a representative GF-2 example. Among the deep learning-based methods, LightNet struggles with spectral fidelity, showing a significant loss of detail. Other methods, including CTINN, SFIIN, MDCUN, and PAPS, demonstrate slight improvements; however, their results are affected by blurred rooftop edges, particularly in the road areas. These results provide strong quantitative evidence that CDFAN effectively addresses the entropy degradation problem often observed in traditional spatial-domain fusion approaches.

### 4.5. Ablation Experiments and Discussion

#### 4.5.1. Computational Efficiency Analysis

**Figure 9:**
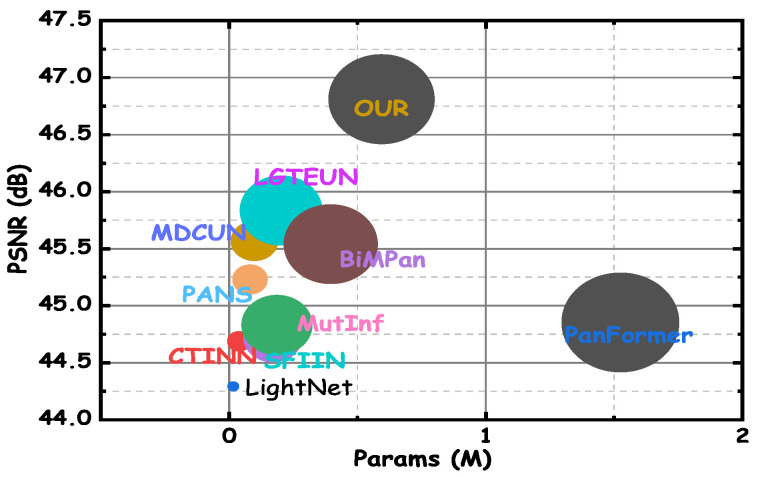
Comparisons of params (X-axis) and PSNR (Y-axis).

Figure 9 shows the balance between computational cost and performance for all algorithms, with CDFAN achieving substantial performance improvements despite requiring additional computational resources due to advanced modules like MDIA, SMCE, and DWT. The ablation study in Table 6 demonstrates that each module enhances performance without significantly increasing the number of parameters. For example, when MDIA is used alone (index 1), the model achieves a PSNR of 45.0148 with 0.2754 million parameters. Adding SMCE (index 2) and DWT (index 3) increases the PSNR to 45.8971 with only a modest increase in parameters to 0.2927 million. The final model, including all modules (index 4), achieves the highest PSNR of 46.8108, with a slight increase in parameters to 0.2988 million. These results confirm that while the inclusion of advanced processing mechanisms increases the computational cost, the performance gains—especially in terms of PSNR, SSIM, and SAM—justify the added computational load, making CDFAN a highly effective solution for high-resolution remote sensing applications.

#### 4.5.2. Effectiveness of the DWT Decomposition

**Figure 10:**
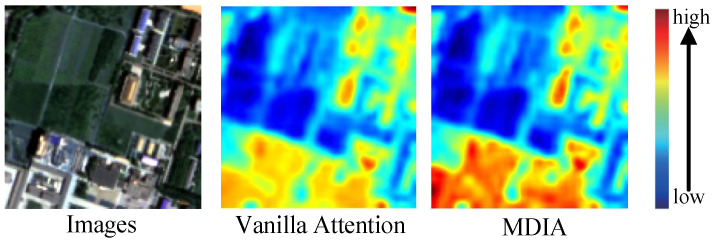
Different heat maps from attention-varied MDIA.

To address the concern regarding potential artifacts or detail loss introduced by DWT, we conducted an ablation study to evaluate its actual impact within our framework. As shown in Table 6, removing DWT from the pipeline (index 3) results in a noticeable drop in performance metrics: the PSNR decreases from 46.8108 to 45.8971, and SAM increases from 1.2490 to 1.4354, suggesting increased spectral distortion and reduced spatial fidelity. While it is true that traditional DWT-based models may introduce artifacts due to the coarse decomposition of frequency components, our integration of DWT into the MDIA module mitigates this issue by guiding the attention mechanism toward high-frequency components. As shown in the attention heatmaps (Figure 10), MDIA with DWT more effectively highlights critical spatial features—such as rooftops and edges—compared to vanilla attention. These results demonstrate that, in the context of our model design, DWT not only avoids generating noticeable artifacts but also contributes significantly to preserving fine-grained textures and enhancing feature localization.

#### 4.5.3. Effectiveness of MDIA

To evaluate the specific contribution of the MDIA module, we conducted both qualitative and quantitative comparisons. As shown in Table 6, removing MDIA (index 2) leads to a noticeable performance drop. Specifically, the PSNR decreases from 46.8108 to 43.5478, while the SAM increases from 1.2490 to 1.7154, indicating increased spectral distortion and a loss of fine-grained details. The absence of MDIA also results in blurred textures, particularly in areas with high structural complexity, such as rooftops, as demonstrated in Figure 10 (case 1). This focused attention helps to preserve important high-frequency features, improving the fusion of spatial and spectral information. Overall, these results confirm that MDIA is vital for maintaining the high image fidelity of the fused output, improving both visual quality and performance metrics by enhancing the model’s ability to focus on relevant image features.

#### 4.5.4. Effectiveness of SMCE

To further examine the effectiveness of SMCE, we isolate its core component—Expert Feature Compensator (EFC)—and evaluate different convolutional configurations to substitute the EFC. Initially, the EFC used a set of multi-scale convolutions. However, to optimize the fusion process, we replaced this with combinations of 3 × 3, 3 × 3, and 5 × 5, and 3 × 3, 5 × 5, and 7 × 7 convolutions, as shown in Table 7. The results in Table 7 demonstrate that as we incorporate larger kernel sizes and more combinations, the model’s performance improves. When only the 3 × 3 kernel is used (index 1), the PSNR reaches 45.5014. By adding a 5 × 5 kernel (index 2), the performance slightly improves to 45.5789, indicating the positive effect of using multiple kernel sizes for better feature extraction. The combination of 3 × 3, 5 × 5, and 7 × 7 kernels (index 3) yields an even better PSNR of 45.6185, suggesting that a broader range of spatial features are captured with these multi-scale convolutions. Finally, when the full EFC module is included (index 4), which uses all the previous configurations with the EFC mechanism, the PSNR reaches its highest value of 46.8108. This demonstrates that the EFC with these multi-scale convolutions significantly contributes to the performance improvement by better compensating for fine spatial details.

## 5. Conclusions

In this study, we proposed an innovative pansharpening framework to address the fundamental challenge of simultaneously enhancing the spatial and spectral resolution of LRMS images. The proposed Cross-Domain Fusion Attention Network (CDFAN) leverages the spectral richness of LRMS images and the fine spatial details of PAN images, achieving high-quality HRMS reconstructions. The architecture is built upon two key components: the Multi-Domain Interactive Attention (MDIA) module and the Spatial Multi-Scale Enhancement (SMCE) module. The MDIA module employs DWT to decompose the PAN image into multiple frequency sub-bands, enabling efficient separation and modeling of high- and low-frequency components. By constructing a multi-domain attention mechanism across wavelet and spatial representations, this module allows for effective fusion guided by information-aware cross-modal interactions, thereby enhancing the preservation of mutual information and reducing spatial–spectral redundancy. The SMCE module complements this by utilizing multi-scale convolution operations and an Expert Feature Compensator, which dynamically balances global and local contributions to ensure optimal feature weighting. This design significantly improves the entropy structure of the output, ensuring both fine-grained texture retention and structural integrity in the PAN image. Extensive experimental results across multiple benchmark datasets demonstrate that CDFAN consistently outperforms existing state-of-the-art methods, achieving notable improvements in both information-theoretic metrics (e.g., QNR, SAM) and perceptual quality. However, the proposed framework presumes accurate spatial alignment between PAN and LRMS inputs—a condition often unmet in real-world applications. In the presence of misregistrations, performance may degrade due to the lack of intrinsic alignment correction. Future extensions could incorporate entropy-aware objectives and content-adaptive denoising modules to enhance robustness under practical deployment conditions.

## Figures and Tables

**Table 1:** Overview of the three benchmark datasets.

Satellite	GaoFen-2	WorldView-2	WorldView-3
Step size	52	18	8
Reduced-resolution image	1036	1012	910
pairs for training			
Reduced-resolution image	136	145	144
pairs for testing			
Full-resolution image	120	120	120
pairs for testing			
Radiometric resolution	10	11	11
Resolution—MS	4 m	2 m	1.2 m
Spatial size—PAN	32×32	32×32	32×32
Spatial size—MS	128×128	128×128	128×128
Resolution—PAN	1 m	0.5 m	0.3 m
Scale ratio	4	4	4

**Table 2:** Quantitative comparison of different methods on the WorldView-3 dataset. The best values and the second-best values are, respectively, highlighted by red and blue colors.

Method	WorldView-3				
	PSNR↑	SSIM↑	Q8↑	SAM↓	ERGAS↓
PanFormer_22_	30.4772	0.9368	0.9316	3.8503	3.1830
CTINN_22_	31.8564	0.9518	0.9460	3.7815	2.7421
LightNet_22_	32.0018	0.9525	0.9472	3.6612	2.6853
SFIIN_22_	31.6587	0.9492	0.9435	3.7357	2.8016
MutInf_22_	31.8298	0.9523	0.9469	3.6440	2.7526
MDCUN_22_	31.2978	0.9429	0.9363	3.7873	2.9295
LGTEUN_23_	32.2188	0.9545	0.9494	3.4664	2.6286
SSDBPN_23_	31.0621	0.9434	0.9379	3.9649	3.0059
BiMPan_23_	32.2850	0.9565	0.9518	3.4549	2.5821
PAPS_24_	32.2540	0.9550	0.9497	3.4606	2.6127
CDFAN	32.5080	0.9579	0.9537	3.43091	2.5389

**Table 3:** Quantitative comparison of different methods on the WorldView-2 dataset. The best values and the second-best values are, respectively, highlighted by red and blue colors.

Method	WorldView-2				
	PSNR↑	SSIM↑	Q4↑	SAM↓	ERGAS↓
PanFormer_22_	41.3581	0.9731	0.8236	1.3808	1.0617
CTINN_22_	41.2015	0.9735	0.8149	1.4095	1.0880
LightNet_22_	41.5589	0.9739	0.8220	1.3579	1.0382
SFIIN_22_	41.9489	0.9752	0.8108	1.3121	1.0084
MutInf_22_	41.9522	0.9760	0.8258	1.3006	1.0153
MDCUN_22_	42.3351	0.9772	0.8370	1.2376	0.9638
LGTEUN_23_	42.6240	0.9782	0.8401	1.2089	0.9282
BiMPan_23_	42.2729	0.9771	0.8380	1.2433	0.9649
PAPS_24_	41.6371	0.9746	0.8241	1.3407	1.0316
CDFAN	42.7719	0.9789	0.8448	1.1747	0.9159

**Table 4:** Quantitative comparison of different methods on the GaoFen-2 dataset. The best values and the second-best values are, respectively, highlighted by red and blue colors.

Method	GaoFen-2				
	PSNR↑	SSIM↑	Q4↑	SAM↓	ERGAS↓
PanFormer_22_	44.8540	0.9805	0.8865	1.5527	1.3334
CTINN_22_	44.2942	0.9784	0.8716	1.6788	1.4148
LightNet_22_	44.6876	0.9787	0.8741	1.5986	1.3510
SFIIN_22_	44.7248	0.9802	0.8721	1.6043	1.3361
MutInf_22_	44.8305	0.9800	0.8836	1.5871	1.3394
MDCUN_22_	45.5677	0.9825	0.8915	1.4439	1.2249
LGTEUN_23_	45.8364	0.9840	0.8973	1.4152	1.1824
BiMPan_23_	45.5398	0.9822	0.8928	1.4324	1.2357
PAPS_24_	45.2308	0.9815	0.8836	1.5126	1.2710
CDFAN	46.8108	0.9868	0.9117	1.2490	1.0588

**Table 5:** Average results at full resolution. The best values and the second-best values are, respectively, highlighted by red and blue colors.

Method	WorldView-3			GaoFen-2		
	$D_{\lambda }$↓	$D_{s}$↓	QNR↑	$D_{\lambda }$↓	$D_{s}$↓	QNR↑
PanFormer_22_	0.0191	0.0416	0.9400	0.0670	0.1806	0.7639
CTINN_22_	0.0123	0.0442	0.9440	0.0697	0.1965	0.7469
LightNet_22_	0.0185	0.0282	0.9539	0.0661	0.2126	0.7358
SFIIN_22_	0.0198	0.0352	0.9458	0.0687	0.1876	0.7557
MutInf_22_	0.0163	0.0420	0.9423	0.0755	0.1762	0.7613
MDCUN_22_	0.0747	0.1673	0.7708	0.0712	0.1938	0.7712
LGTEUN_23_	0.0162	0.0310	0.9532	0.0696	0.1981	0.7457
BiMPan_23_	0.0298	0.0305	0.9463	0.0644	0.1419	0.8120
PAPS_24_	0.0262	0.0581	0.9181	0.0663	0.1575	0.7861
CDFAN	0.0289	0.0293	0.9539	0.0681	0.1409	0.8121

**Table 6:** Comparative assessment of module ablations on GF-2 dataset.

Index	MDIA	SMCE	DWT	PSNR	SSIM	SAM	Params
1	✔		✔	45.0148	0.9798	1.5897	0.2754
2		✔	✔	43.5478	0.9678	1.7154	0.2601
3	✔	✔		45.8971	0.9838	1.4354	0.2927
4	✔	✔	✔	46.8108	0.9868	1.2490	0.2988

**Table 7:** Ablation study of SMCE on the GF-2 dataset.

Index	PSNR	SSIM	SAM	Params
3 × 3	45.5014	0.9801	1.5597	0.2837
3 × 3 and 5 × 5	45.5789	0.9819	1.5521	0.2846
3 × 3 and 5 × 5 and 7 × 7	45.6185	0.9821	1.5478	0.2854
EFC	46.8108	0.9868	1.2490	0.2988

## Data Availability

Web links to the datasets analyzed during the current study are inserted as footnotes in the article.
